# Relationship of the SITLESS intervention on medication use in community-dwelling older adults: an exploratory study

**DOI:** 10.3389/fpubh.2023.1238842

**Published:** 2023-11-16

**Authors:** Ruben Viegas, Filipa Alves da Costa, Romeu Mendes, Manuela Deidda, Emma McIntosh, Oriol Sansano-Nadal, Juan Carlos Magaña, Dietrich Rothenbacher, Michael Denkinger, Paolo Caserotti, Mark A. Tully, Marta Roqué-Figuls, Maria Giné-Garriga

**Affiliations:** ^1^Faculty of Pharmacy, iMED, Research Institute for Medicines, University of Lisbon, Lisbon, Portugal; ^2^Egas Moniz Center for Interdisciplinary Research (CiiEM), Egas Moniz School of Health and Science, Almada, Portugal; ^3^EPIUnit - Instituto de Saúde Pública, Universidade do Porto, Porto, Portugal; ^4^ACES Douro I – Marão e Douro Norte, Administração Regional de Saúde do Norte, Vila Real, Portugal; ^5^Health Economics and Health Technology Assessment (HEHTA), Institute of Health and Wellbeing (IHW), University of Glasgow, Glasgow, United Kingdom; ^6^Faculty of Psychology, Education and Sport Sciences Blanquerna, Universitat Ramon Llull, Barcelona, Spain; ^7^School of Health and Sport Sciences (EUSES), Rovira i Virgili University, Tarragona, Spain; ^8^Institute of Epidemiology and Medical Biometry, Ulm University, Ulm, Germany; ^9^Institute for Geriatric Research at Agaplesion Bethesda Clinic and Geriatric Centre, Ulm University Medical Centre, Ulm, Germany; ^10^Department of Sports Science and Clinical Biomechanics, Center for Active and Healthy Ageing, University of Southern Denmark, Odense, Denmark; ^11^School of Medicine, University of Ulster, Londonderry, United Kingdom; ^12^Iberoamerican Cochrane Centre, Biomedical Research Institute Sant Pau (IIB Sant Pau), CIBER Epidemiología y Salud Pública (CIBERESP), Barcelona, Spain; ^13^Faculty of Health Sciences Blanquerna, Universitat Ramon Llull, Barcelona, Spain

**Keywords:** physical activity, older adults, medication use, intervention, sedentary behavior, primary health care

## Abstract

**Background:**

Sedentary behavior (SB) and physical activity (PA) interventions in older adults can improve health outcomes. Problems related with aging include prevalent comorbidity, multiple non-communicable diseases, complaints, and resulting polypharmacy. This manuscript examines the relationship between an intervention aiming at reducing SB on medication patterns.

**Method:**

This manuscript presents a local sub-analysis of the SITLESS trial data on medication use. SITLESS was an exercise referral scheme (ERS) enhanced by self-management strategies (SMS) to reduce SB in community-dwelling older adults. We analyzed data from the ERS + SMS, ERS and usual care (UC) groups. Patient medication records were available at baseline and at the end of the intervention (4-month period) and were analyzed to explore the effect of SITLESS on medication patterns of use.

**Result:**

A sample of 75 participants was analyzed, mostly older overweight women with poor body composition scores and mobility limitations. There was a significant reduction of 1.6 medicines (SD = 2.7) in the ERS group (*p* < 0.01), but not in the UC or ERS + SMS groups. Differences were more evident in medicines used for short periods of time.

**Conclusion:**

The findings suggest that an exercise-based program enhanced by SMS to reduce SB might influence medication use for acute conditions but there is a need to further investigate effects on long-term medicine use in older adults.

## 1. Introduction

Increased longevity and improved health at older ages seen globally present significant challenges such as older adults having a higher prevalence of multimorbidity that often results in simultaneous treatment with multiple drugs (1). Polypharmacy (i.e., using five or more medications) (2) may increase the number of drug-related problems such as adverse effects and interactions, and is frequently associated with an increased use of potentially inappropriate medications (PIMs). Polypharmacy could have negative associations on long-term physical and cognitive functions (3–5).

Higher levels of physical activity (PA; occupational, commuting, leisure-time and household chores) have been significantly associated with a decreased risk of medicine use even after adjusting for sex, age, and economic status (6, 7). In a previous study, both medication use, and lack of PA were strongly associated with poor self-reported health status in older adults (8). More recently, sedentary behavior (SB; any waking activity in a sitting, reclining, or lying posture where energy expenditure is <1.5 metabolic equivalents) has been also considered an important determinant of health-related quality of life in older adults (9). Other studies conducted in a similar population showed that the likelihood of being categorized as highly sedentary increased with poor self-rated health, being overweight or obese, having a history of smoking, and multiple medication use (10, 11). An association between both sedentary time and cardiometabolic risk factors (such as metabolic syndrome and obesity) in older adults and between sedentary time and increased medication use have also been suggested (12).

However, evidence on whether a program aimed at reducing SB and increasing PA may help decrease medication use is yet to be established. This sub-study within the SITLESS Trial evaluated the relationship of an exercise referral scheme (ERS) enhanced by self-management strategies (SMS) aimed at reducing SB—SITLESS intervention- (13) and targeting community-dwelling older adults, on medication use compared to the ERS alone and usual care (UC) in a subsample of the population. Therefore, this study aims to understand how PA interventions in older adults can influence medication use. The main hypothesis established was that increased physical activity is associated with decreased medication needs. A secondary hypothesis was that the medication group that could potentially be reduced would be the medicines that affected the Central Nervous System.

## 2. Materials and methods

### 2.1. Participants

This research is a sub analysis of the SITLESS trial data. Briefly, SITLESS was a multi-center pragmatic three-armed randomized controlled trial conducted in 4 European countries. The purpose of the initial study was to assess the short and long-term effectiveness (and cost-effectiveness) of ERS program enhanced by SMS to reduce SB and increase PA and other health outcomes in community-dwelling European older adults (≥65 years old). The original SITLESS study was designed as RCT, where participants were randomly allocated into a usual care group (UC), a physical activity program alone (ERS), and the same physical activity program + Self-Management Strategies (ERS + SMS). Participants were recruited through primary care centers and posters, flyers, newspapers, radio broadcasts and social media outlets were also used to advertise the study as additional recruitment strategies, considering as eligibility criteria (1) aged 65 years or above; (2) community-dwelling; (3) able to walk without the help of another person for at least 2 min with or without a walking aid; (4) have no major physical limitations as shown by a score on the Short Physical Performance Battery (SPPB) of 4 or above [35]; (5) insufficiently active as determined by the following screening question: “Do you perform regular physical activity (PA) for at least 30 min five or more days of the week (referring only to PA that makes the participant become out of breath while doing it or such that it does not allow him/her to maintain a conversation while doing the activity; do not count regular walking)”; and/ or (6) report spending long periods of time in SB by answering affirmatively to the question: “For most days, do you feel you sit for too long (6–8 h or more a day)? Some examples might include when watching TV, working at the computer/laptop or when doing sitting-based hobbies such as sewing.” The sample was estimated aiming to detect a moderate effect size of 30 daily counts per minute (CPM) in a two-sided test, at a power of 80% and an α of 0.05, a common standard deviation of 139 of the mean and a 24% dropout rate. Included participants were insufficiently active (<150 min/week) and/or reported being highly sedentary (>6 h in SB). More information about the SITLESS study can be found elsewhere (13).

The ERS was a 16-week program with two 60-min sessions per week, including aerobic, balance and resistance training. The ERS + SMS participants received, concurrently to the 16-week PA program offered to ERS participants, a face-to-face visit, six-group sessions and four telephone calls. Participants allocated to the usual UC were offered two health advice meetings with general recommendations on healthy lifestyle.

For this sub-study, we used a convenience sample of 75 of 337 older adults from Barcelona, who had information on the number of medicines at baseline and post-intervention (month 4) and further explored a sub-sample of 20, who had specific information on the active ingredients being used on both these periods. For this analysis, besides analyzing the 3 groups separately, we explored possible combinations of the groups to identify relationships: (UC + ERS) and [ERS + (ERS + SMS)].

### 2.2. Data collection

The current study complements previous research by exploring whether the SITLESS intervention led to a change in overall medication use after the intervention (4-month follow up). All data were collected between 2016 and 2017. Medicines were identified from the medication database available and described using International Nonproprietary Names (INN). Besides prescription medicines, over the counter medicines and supplements, such as vitamins, were also considered for this analysis. The number and type of current medications were obtained through an individual interview. Information about medicines included the medicine brand name that was translated to INN, dosage, and daily frequency.

### 2.3. Statistical analysis

The main participant characteristics are presented descriptively as mean and standard deviation (SD) for continuous variables or number and percentage for categorical variables. Covariates used to describe the sample included sex, age, Body Mass Index (BMI), waist and hip circumferences (WC and HC) and the Short Physical Performance Battery (SPPB) score. *T*-tests are used to identify changes in continuous variables from different groups between baseline and post-intervention. Other statistical methods such as a linear regression could not be employed due to the limited sample available for this sub-study.

In addition, a mixed-method approach was used to explore in more detail the pharmacotherapeutic groups whose consumption decreased following the SITLESS intervention. These are presented descriptively, and changes explained using as framework the mechanistic mode of action of each class and focusing on common characteristics.

All statistical analyses were performed using IBM SPSS Statistics 26 (SPSS, Inc., an IBM Company, Chicago, IL, United States) and the significance level was set at *p* < 0.05.

## 3. Results

Participants’ characteristics at baseline are presented in Table 1. The sample was composed mostly of older overweight adults (77% female). The waist to hip ratio was on average 0.91 (SD = 0.09) which indicates a high health risk. Participants presented in average 3.2 (SD = 2.1) comorbidities: 52% had high blood pressure (UC 61%; ERS 46%; ERS + SMS 50%), 50% had arthritis (UC 48%; ERS 58%; ERS + SMS 42%), 31% had obesity (UC 13%; ERS 39%; ERS + SMS 39%), 20% chronic pain (UC 17%; ERS 19%; ERS + SMS 23%), 19% diabetes (UC 4%; ERS 19%; ERS + SMS 31%), 15% depressive disorders (UC 26%; ERS 12%; ERS + SMS 8%) and 12% anxiety disorders (UC 9%; ERS 12%; ERS + SMS 15%). The average blood pressure for the sample was 134.4 (SD = 15.9)/80.0 (SD = 12.0). Regarding smoking habits, 10% were active smokers, 23% ex-smokers and 67% non-smokers. Smokers had been smoking for 30.6 years (SD = 17.7) with an average of 13.4 (SD = 7.9) cigarettes per day. The SPPB score indicates that the sample had one or more mobility limitations.

**Table 1:** Sample demographic and descriptive characteristics at baseline.

	Total sample (*n* = 75) (77.3% female)	UC (*n* = 23) (82.6% female)	ERS (*n* = 26) (76.9% female)	ERS + SMS (*n* = 26) (73.1% female)	(UC + ERS) (*n* = 49) (79.6% female)	[ERS + (ERS + SMS)] (*n* = 52) (75.0% female)
Age	75.7 (SD = 6.5)	75.5 (SD = 6.7)	76.6 (SD = 7.0)	75.5 (SD = 6.7)	75.9 (SD = 6.5)	76.0 (SD = 6.8)
BMI	29.6 (SD = 5.2)	28.6 (SD = 4.1)	30.2 (SD = 4.5)	29.8 (SD = 6.6)	29.5 (SD = 4.3)	30.0 (SD = 5.6)
W/H ratio	0.9 (SD = 0.1)	0.9 (SD = 0.1)	0.9 (SD = 0.1)	0.9 (SD = 0.1)	0.9 (SD = 0.1)	0.9 (SD = 0.1)
SPPB	8.8 (SD = 2.5)	8.7 (SD = 2.6)	8.1 (SD = 2.9)	8.7 (SD = 2.6)	8.9 (SD = 2.4)	8.4 (SD = 2.7)
Comorbidities	3.2 (SD = 2.1)	3.3 (SD = 2.0)	3.3 (SD = 2.3)	3.1 (SD =1.9)	3.3 (SD = 2.2)	3.2 (SD = 2.1)
Daily step count	4716.9 (SD = 1960.3)	5262.4 (SD = 1998.9)	4175.3 (SD = 1903.8)	4756.9 (SD = 1921.7)	4694.1 (SD = 2003.6)	4478.2 (SD = 1915.3)

UC, Usual care; ERS, Exercise Referral Scheme; ERS + SMS, Exercise Referral Schemes and Self-Management Strategies; BMI, Body Mass Index; WC, Waist circumference; W/H ratio, Waist to hip ratio; SPPB, Short Physical Performance Battery.

Differences in medicine use were observed in the entire sample and in all subgroups (Table 2) between baseline and after the intervention. However, significant reductions in medicine use were most visible and significant only in the ERS alone group or when collapsing this group with the ERS + SMS group.

**Table 2:** Changes in medicine use in the different groups.

Number of medicines	Baseline	End of intervention	Observed change
Total sample (*n* = 75)	5.7 (SD = 3.8)	4.6 (SD = 3.2)	−1.1 (SD = 2.7), *p* < 0.01
UC (*n* = 23)	4.8 (SD = 3.8)	3.7 (SD = 2.7)	−1.1 (SD = 2.8), *p* = 0.08
ERS alone (*n* = 26)	6.3 (SD = 4.1)	4.7 (SD = 3.5)	−1.6 (SD = 2.7), p < 0.01
ERS + SMS (*n* = 26)	5.9 (SD = 3.5)	5.4 (SD = 3.3)	−0.5 (SD = 2.7), *p* = 0.30
(UC + ERS) (*n* = 49)	5.5 (SD = 4.0)	4.2 (SD = 3.2)	−1.3 (SD = 2.7), p < 0.01
[ERS + (ERS + SMS)] (*n* = 52)	6.1 (SD = 3.8)	5.0 (SD = 3.4)	−1.1 (SD = 2.7), p < 0.01

UC, Usual care; ERS, Exercise Referral Scheme; ERS + SMS, Exercise Referral Schemes and Self-Management Strategies.

The subgroup of patients that had a complete medication record (*n* = 20) including active ingredients and dosages was analyzed to better understand the type of changes that might have happened (Table 3). Most changes occurred in medicines that were used for acute situations (e.g., vitamins, eye drops or anti-inflammatories).

**Table 3:** Detailed medication analysis (*n* = 20).

Patient ID	Group^*^	Initial medicine use (and number of medicines)	Post-intervention medication use (and number of medicines)	Difference in medicine number	Detail of difference in medicines
1	1	Brimonidine eye drops 1,5 g 1D; Dexamethasone eye drops 1 g 1D; Enalapril 20 mg 2D; Vitamin E 5,000 UI/10 mg 1D (n = 4)	Enalapril 20 mg 2D (n = 1)	−3	Two formulations removed were eye drops and another was a vitamin supplement.
2	1	Tramadol 50 mg 1D (n = 1)	Bisoprolol 2,5 mg; Omeprazole 20 mg 1D; Tramadol 50 mg 1D (n = 3)	+2	Inclusion of Bisoprolol and Omeprazole.
3	1	Bisoprolol 5 mg 1D; Losartan 50 mg 1D (n = 2)	Dabigatran 150 mg 1D; Omeprazole 20 mg 1D (n = 2)	0	Hypertension medication was removed, and dabigatran and omeprazole added.
4	2	Lisinopril 20 mg 1D; Metamizole 5 mg 0,5D; Verapamil 240 mg 1D; Vitamin D 1.5 mL 1D (n = 4)	Lisinopril 20 mg 1D; Verapamil 240 mg 1D; Vitamin D 1.5 mL 1D (n = 3)	−1	Metamizole was removed.
5	3	Acenocumarol20 mg 1D; Atorvastatin 20 mg1D; Bisoprolol 2.5 mg 3D; Budesonide/formoterol 80/4,5 mcg 4D; Exenatide 10 mcg 2D; Furosemida40mg 1D; Linagliptin 5 mg 1D; Omeprazole 20 mg 1D; Spironolactone 60 mg 2D (n = 9)	Acenocoumarin 4 mg 1D; Atorvastatin 20 mg 1D; Bisoprolol 2,5 mg 3D; Budesonide/formoterol 80/4,5 mcg 4D; Diltiazem 60 mg 2D; Exenatide 10 mcg 2D; Furosemide 40 mg 2D; Insulin 3 mL 10 units 1D; Linagliptin 5 mg 1D; Omeprazole 20 mg 1D; Paracetamol 1 g 1D (n = 11)	+2	Paracetamol and insulin were added to diabetes therapy. Spironolactone was removed and diltiazem was added.
6	3	Acenocoumarin 4 mg 1D; Acetylsalicylic acid 100 mg 1D; Amiodarone 200 mg 1D; Atorvastatin 40 mg 1D; Enalapril 5 mg 1D; Tiotropium 18 mcg 1D (n = 6)	Amiodarone 200 mg 1D; Atorvastatin 40 mg 1D; Enalapril 5 mg 1D; Tiotropium 18 mcg 1D (n = 4)	−2	Removal of aspirin and acenocoumarin.
7	2	Calcifediol 0,266 mg 30D; Calcium carbonate 500 mg 2D; Amiloride 5 mg 1D; Hydrochlorothiazide 50 mg 1D (n = 4)	Calcium carbonate 500 mg 2D; Calcifediol 0,266 mg 30D; Amiloride 5 mg 1D; Hydrochlorothiazide 50 mg 1D (n = 4)	0	No changes.
8	2	Acetylsalicylic acid 100 mg 1D; Bimatoprost eye drops 0.3 mg 1D; Bisoprolol 5 mg 1D; Glycerol trinitrate 5 mg 1D; Olmesartan 40 mg 1D; Omeprazole 20 mg 1D; *Serenoa repens* 160 mg 2D; Simvastatin 20 mg 1D; Vitamin D 0.3 mg 30D (n = 9)	Acetylsalicylic acid 100 mg 1D; Bisoprolol 5 mg 1D; Glycerol trinitrate 5 mg 1D; Olmesartan 40 mg 1D (n = 4)	−5	Stopped omeprazole, a natural supplement of “*Serenoa repens*,” vitamin D, simvastatin and bimatoprost eye drops.
9	3	Acetylsalicylic acid 100 mg 1D; Alopurinol 300 mg 1D; Atenolol 100 mg 1D; Atorvastatin 40 mg 1D; Fesoterodine 8 mg 1D; Metformin 850 mg 1D; Omeprazole 20 mg 0,5D; Paracetamol 500 mg 4D; Pioglitazone 30 mg 1D; Pregabalin 150 mg 1D; Valsartan 320 mg 1D; Vitamin D 0,266 mg 30D (n = 12)	Acetylsalicylic acid 100 mg 1D; Alopurinol 300 mg 1D; Alprazolam 0,25 mg 2D; Atenolol 100 mg 1D; Atorvastatin 40 mg 1D; Fesoterodine 8 mg 1D; Metformin 850 mg 3D; Omeprazole 20 mg 0,5D; Pioglitazone 30 mg 1D; Pregabalin 150 mg 1D; Valsartan 320 mg 1D; Vitamin D 0,266 mg 30D (n = 12)	0	Stopped paracetamol but alprazolam was added.
10	3	Enalapril 20 mg 1D; Hydrochlorothiazide 12,5 mg 1D; Omeprazole 20 mg 1D (n = 3)	Enalapril 20 mg 1D; Lorazepam 1 mg 1D; Omeprazole 20 mg 1D; Sertraline 100 mg 1D; Vitamin D 2,5 mL 30D (n = 5)	+2	Addition of sertraline, lorazepam, and vitamin D. Removal of hydrochlorothiazide.
11	2	Enalapril 5 mg 1D; Paracetamol 500 mg 3D; Tamsulosin 0,4 mg 1D; Vitamin B 1,000 mcg 30D (n = 4)	Enalapril 5 mg 1D; Tamsulosin 0,4 mg 1D; Vitamin B 1,000 mcg 30D (n = 3)	−1	Stopped paracetamol.
12	1	Atorvastatin 20 mg 1D; Dorzolamide 20 mg/mL 2D; Latanoprost 50 mcg/mL 1D; Timolol 5 mg 7 mL 2D (n = 4)	Atorvastatin 20 mg 1D; Dorzolamide 20 mg/mL 2D; Latanoprost 50 mcg/mL 1D (n = 3)	−1	Timolol (eye drops) was removed.
13	3	Alopurinol 300 mg 1D; Digoxin 0,25 mg 1D; Enalapril 5 mg 0,5D; Ferro glycine 100 mg 1D; Furosemide 40 mg 1D; Hydroxycarbamide 500 mg 2D; Ketoconazole 100 mL 3D; Paracetamol 1 g 2D; Salmeterol 25 mcg 2D; Spironolactone 25 mg 1D; Vitamin B 1,000 mcg 30D (n = 11)	Alopurinol 300 mg 1D; Digoxin 0,25 mg 1D; Enalapril 5 mg 0,5D; Ferro glycine 100 mg 1D; Furosemide 40 mg 1D; Hydroxycarbamide 500 mg 2D; Ketoconazole 100 mL 3D; Omeprazole 20 mg 1D; Paracetamol 1 g 2D; Salmeterol 25mcg 2D; Spironolactone 25 mg 1D; Vitamin B 1,000 mcg 30D (n = 12)	+1	Omeprazole was added.
14	1	Acetylsalicylic acid 100 mg 1D; Budesonide 200 mcg 4D; Calcium Carbonate 500 mg 2D; Galantamine 16 mg 1D; Hydrochlorothiazide 12,5 mg 1D; Lormetazepam 2 mg 1D; Losartan 50 mg 1D; Omeprazole 20 mg 1D; Paracetamol 1 g 1D; Salmeterol 50 mcg 2D; Simvastatin 10 mg 1D; Vitamin D 400ui 1D (n = 12)	Acetylsalicylic acid 100 mg 1D; Lormetazepam 2 mg 1D; Losartan 50 mg 1D; Omeprazole 20 mg 1D; Paracetamol 1 g 1D; Salmeterol 50 mcg 2D; Simvastatin 10 mg 1D (n = 7)	−5	Budesonide, Galantamine, Calcium, Vitamin D and Hydrochlorothiazide were removed.
15	2	Atorvastatin 10 mg 1D; Bisoprolol 2,5 mg 1D; Citalopram 10 mg 1D; Diazepam 2,5 mg 1D; Hydrochlorothiazide 12,5 mg 1D; Lisinopril 20 mg 1D; Pantoprazole 20 mg 1D; Warfarin 1 mg 1D (n = 8)	Atorvastatin 10 mg 1D; Bisoprolol 2,5 mg 1D; Diazepam 2,5 mg 1D; Lisinopril 20 mg 1D; Pantoprazole 20 mg 1D; Warfarin 1 mg 1D (n = 6)	−2	Hydrochlorothiazide and Citalopram were removed.
16	3	Acetylsalicylic acid 150 mg 1D; Amitriptyline 25 mg 1D; Bisoprolol 5 mg 1D; Fluoxetine 20 mg 1D; Flupenthixol + Melitracen 0,5 + 3 mg 1D; Hydrochlorothiazide 12,5 mg 1D; Ezetimibe 10 mg 1D; Metformin 850 mg 1D; Omeprazole 20 mg 1D; Quetiapine 25 mg 1D; Rosuvastatin 20 mg 1D; Valsartan 320 mg 1D (n = 12)	Acetylsalicylic acid 150 mg 1D; Amitriptyline 25 mg 1D; Bisoprolol 5 mg 1D; Clonazepam 2,5 mg/mL 1D; Duloxetine 60 mg 1D; Ezetimibe 10 mg 1D; Levodopa 200/50 mg 3D; Metformin 850 mg 1D; Omeprazole 20 mg 1D; Rosuvastatin 20 mg 1D; Valsartan 320 mg 1D (n = 11)	−1	Levodopa and Duloxetine were added. Flupentixol, Fluoxetine and Hydrochlorothiazide were removed.
17	2	Duloxetin 100 mg 1D; Lorazepam 0,5D; Otilonium bromide 40 mg 1D; Paracetamol 650 mg 1D (n = 4)	Duloxetin 100 mg 1D; Lorazepam 0,5D; Paracetamol 650 mg 1D; Vitamins 1D (n = 4)	0	Otilonium bromide was removed and vitamins were added.
18	2	Enalapril 50 mg 1D; Omeprazole 20 mg 1D (n = 2)	Enalapril 50 mg 1D (n = 1)	−1	Omeprazole was removed.
19	3	Lisinopril 5 mg 1D (n = 1)	Lisinopril 5 mg 1D; Sulpiride 200 mg 2D (n = 2)	+1	Sulpiride was added.
20	1	Acetylsalicylic acid 100 mg 1D; Omeprazole 20 mg 1D (n = 2)	Acetylsalicylic acid 100 mg 1D; Omeprazole 20 mg 1D; Paracetamol 500 mg 3D (n = 3)	+1	Paracetamol was added.

*Group 1=Usual Care; Group 2=Exercise Referral Scheme only; Group 3=Exercise Referral Schemes and Self-Management Strategies.

## 4. Discussion

This study contributes to the evidence base on medication use impacts of interventions aimed at reducing SB and promoting PA in community-dwelling older adults. It was hypothesized that the SITLESS intervention would contribute to a change in medication use patterns. We could observe a reduction for the whole sample and for the ERS groups but could not fully explain all the causes for this reduction. The reduction of medicine burden in older adults can be one solution for the growing problem of polypharmacy, that could have negative associations on long-term physical and cognitive functions (3–5). Exercise interventions could provide a solution for better health outcomes while providing older adults with a longer life and lower medicine burden.

The sample included in this sub analysis showed poor values of body composition (BMI = 29; waist to hip ratio = 0.91; daily step count around 5,000 steps). Interventions targeting SB and PA can lead to improved body composition and might prevent the development of chronic diseases, leading ultimately to lower use of medication by older adults (14, 15). In order to explore this relationship in further detail, more studies should aim to collect movement behavior in different timepoints (preferably with accelerometry), body composition and medication outcomes, and also analyze the interaction between these variables.

The difference in the number of medicines at baseline and after intervention seems to follow a trend of reduction in overall number of medicines taken, as seen in other studies (6). Even though the reduction was seen in the overall sample and in the subgroup ERS alone, the ERS + SMS group did not show a significant reduction. However, when combining these two subgroups [ERS alone + (ERS + SMS)] the change became significant also, suggesting the inability to detect a significant change in all subgroups receiving the main intervention (ERS) could result mainly from the small sample size and not from the intervention *per se*. It is also reasonable to expect longer-term effects of the SMS sessions enhancing the effects of the ERS, thus only captured later. SMS had been shown to be effective in changing behavior (16), thus to evaluate the effects of the SITLESS intervention in medication use, particularly the enhanced approach using SMS, compared to ERS alone or UC we may need a longer follow up. This gains relevance when the average number at baseline indicates polypharmacy (> 5 medicines) in most groups, which follows a growing trend of polypharmacy in the last years (17). Future studies should aim for obtaining medication use data and whenever possible supplement patient-reported information with electronic health records (18, 19).

Due to the limited sample, we could not explore in detail the causes for the change in medicine use. A more robust sample would have allowed to use a regression model that could provide some clues on the reason for the change in medicine use. Medication management is a complex process that involves multiple healthcare professionals and the patient as the main player. Multiple challenges are known to arise when managing polypharmacy (e.g., transition of care, duplication of therapy, etc.) (20). Future studies could consider the continuum in patient centered care and whenever full integration of electronic records is not possible, increasing the number of contact points with the healthcare system, including with the local pharmacy. This could lead to more detailed and robust information to explore the effects of any intervention on medication patterns (21).

Lastly, the mixed-methods approach taken to explore qualitatively the medication classes most associated with decreases in the overall number of medicines can provide some clues on possible short-term effects of interventions targeting SB. We observed that medications used for acute conditions were those more frequently removed, including painkillers. However, to a lower extent, there was also removal of medications whose effectiveness is questionable (e.g., vitamins) or removal of potentially unsafe medications, including interactions (e.g., acenocumarol + aspirin) or potentially inappropriate medications (e.g., antidepressants with fall-inducing potential). Exercise interventions can support older adults in the prevention of falls and fall-related injuries (22, 23). Considering this, paying special attention to medicines that can affect balance or have a fall-inducing potential can have a protective effect on older adults and ultimately reduce fall risk and improve quality of life. In the current study, we could not fully explore changes in therapeutic subgroups, where changes could be expected, such as fall-risk increasing drugs (FRIDs, which include, e.g., drugs causing sedation, e.g., antipsychotics, drugs causing hypotension, e.g., loop diuretics, drugs with anticholinergic effects, e.g., tricyclic antidepressants, drugs causing bradycardia, e.g., antiarrhythmics, and other associated classes), mostly because of the limited sample size of the sub-analysis but also because of the pitfalls in recording of medication data. However, it has been shown that exercise is the single intervention demonstrated to significantly reduce the rate of falls in community-dwelling individuals (24), which is often associated to deprescribing following medication review in multifactorial interventions (25).

In general, the total quantitative difference might be low; however, qualitatively, the changes observed are aligned with pharmaceutical care principles, by focusing on necessity (e.g., pain-killers patient #11 and #4), effectiveness (e.g., supplements patient #8) and safety (e.g., CNS-acting medicine—patient #15 and #16; or interactions—patient #6). These findings suggest that for exploring the full potential of SB interventions on medication patterns, mixed methods are particularly useful as a change in number might not be clinically significant (26). The full potential of such analysis was however not reached as additional information on indication and duration of exposure would be needed for type 2 medication review. In such reviews, it would be possible to identify according to well established criteria, e.g., if removal of proton-pump inhibitors (patient #18) could also be justified by excessive duration. The benefits of type 3 medication review are even higher, but would also imply having additional information on comorbidities and laboratory values; these would allow judging if, e.g., removal of statins (patient #8 and #15) could be justified by effectiveness, as the most recent criteria consider there is no evidence to support the use of statins for primary prevention of cardiovascular disease in diabetes mellitus (27).

## 5. Conclusion

Many of the alterations in medication may be justified not only by the intervention but also by changes in the clinical condition, for which access to biochemical values would be valuable (e.g., to judge if the use of painkillers is done in the absence of tissue damage) and their absence constitutes one of the limitations of this study. A multidisciplinary approach to medication tracking using both data registries from physicians and pharmacists, and information from caregivers could add more accuracy to the reasons for changes in medication use. Another limitation of this study is the limited sample size with information on medicines used compared to the original sample (*n* = 446). This difference in sample size does not allow for a robust interpretation of the available data and only allows this study to provide an exploratory view of this topic. The reduction in the number of medicines could also be biased by the collection of data, as participants might not have recalled all the medicines after the intervention. A thorough data collection for medicines, including those as main variables of the study, could be a first step into increasing accuracy and more associations between exercise and medication use.

Although this retrospective post-hoc analysis showed possible relationships between lifestyle interventions and medication use, the hypothesis established could not be confirmed. We have identified that exercise interventions can have a modest impact in medication use (i.e., number) and identified some potential medicine groups that can be highlighted in future research (e.g., FRIDs, namely those causing sedation, causing hypotension, or with anticholinergic effects). More robust and exhaustive health information systems, strengthened by interprofessional efforts are needed to better capture the impact of lifestyle interventions on medication use patterns.

## Data availability statement

The data analyzed in this study is subject to the following licenses/restrictions: dataset owned by the original article owner. Requests to access these datasets should be directed to MG-G, mariagg@blanquerna.url.edu.

## Ethics statement

The studies involving humans were approved by the Ethics and Research Committee of Ramon Llull University (Fundació Blanquerna, Spain; reference number: 131400IP). The studies were conducted in accordance with the local legislation and institutional requirements. The participants provided their written informed consent to participate in this study.

## Author contributions

RV, FAC, RM, and MG-G: conceptualization and writing — original draft preparation. RV, FAC, MG-G, MR-F, MaD, MiD, and EM: methodology. OS-N, JCM, DR, MaD, MiD, PC, MT, and MG-G: data collection. RV, FAC, and RM: formal analysis. RV, FAC, RM, MaD, EM, OS-N, JCM, DR, MiD, PC, MT, MR-F, and MG-G: writing — review and editing. FAC, RM, and MG-G: supervision. All authors contributed to the article and approved the submitted version.
